# Active Edible Film Based on Chitosan/Gelatin Incorporated with Protein Hydrolysate from Fish Processing Waste and Its Application for Shelf Life Extension of Sun-Dried Snakeskin Gourami (*Trichogaster pectoralis*)

**DOI:** 10.3390/polym18121446

**Published:** 2026-06-10

**Authors:** Chananun Sukha, Phatthira Sakamut, Benjarat Tepsongkroh, Pontree Itkor, Supattra Supawong, Athip Boonsiriwit

**Affiliations:** 1 Department of Food Science and Technology, Thammasat University, 99 Moo18, Phahonyothin Road, Khlong Luang, Pathum Thani 12120, Thailand; chananun4412@gmail.com (C.S.); psutloet@tu.ac.th (P.S.); benjarat@tu.ac.th (B.T.); pontree.itkor@gmail.com (P.I.); supat29@tu.ac.th (S.S.); 2 Center of Excellence in Food Science and Innovation, Thammasat University, 99 Moo18, Phahonyothin Road, Khlong Luang, Pathum Thani 12120, Thailand

**Keywords:** active edible film, chitosan, gelatin, fish protein hydrolysate, antioxidant, snakeskin gourami, sustainable packaging

## Abstract

Large volumes of waste by-products are generated by the continuing growth of the fish production industry. To address this issue, this study valorized the protein hydrolysate extracted from fish waste for use as an active compound in a packaging system. An edible film was developed based on a chitosan/gelatin (CG) matrix, incorporated with fish protein hydrolysate (FPH) extracted from snakeskin gourami processing waste, at concentrations of 0%, 2%, 4%, 6%, and 8%. Introducing FPH into the film’s matrix enhanced the UV protection properties but decreased transparency. The incorporation of 4% FPH significantly increased the film tensile strength and antioxidant capacity by 47.67% and 65.04%, respectively, compared to the control, while concentrations of FPH exceeding 4% had a detrimental effect on film mechanical and antioxidant capacities. 4P-CG was identified as the optimal formulation and applied as an edible coating for sun-dried snakeskin gourami at 4 °C for 14 days. The 4P-CG coating preserved the quality of sun-dried fish by reducing weight loss and inhibiting microbial growth, which extended the shelf life by four days compared to the control (non-coated) and other CG-coated fish. These findings suggest that 4P-CG is a promising, sustainable active packaging solution for enhancing the stability and safety of fatty aquatic related products while contributing to the circular economy through waste reduction.

## 1. Introduction

The global fish production industry is expanding rapidly, generating large volumes of by-products, such as heads, skin, fins, and viscera, which account for up to 90% of total fish weight. These materials are often discarded or diverted to low-value products like fish meals, posing significant environmental challenges [1,2,3]. Valorizing these waste by-products into high-value functional ingredients, such as protein hydrolysates, is a sustainable strategy to mitigate environmental impacts while enhancing economic viability. Typically, protein hydrolysates can be produced from various methods, such as fermentation or enzymatic hydrolysis [2]. Due to the presence of relatively small bioactive peptides, they exhibit potent antioxidant and antimicrobial activities [2,4]. Previous studies reported that protein hydrolysates extracted from fish heads and viscera exhibited strong antioxidant and antibacterial activities against Gram-positive and Gram-negative bacteria [5,6,7], making them promising candidates for applications as functional food ingredients or integration into active food packaging systems.

Snakeskin gourami (*Trichogaster pectoralis*) is a commercially important fish farmed in Southeast Asia, particularly in Thailand, Vietnam, and Malaysia, and is highly valued for its taste and rapid growth. It is commonly processed into dried salted or sun-dried fish for domestic and export markets. However, sun-dried snakeskin gourami is highly susceptible to lipid oxidation and microbial spoilage, leading to rancidity, off odors, and limited shelf life [8,9]. Vacuum packaging extends its shelf life but causes liquid exudation that compromises the product’s sensory appeal while not preventing rancidity [10]. Thus, there is a critical need for innovative packaging technologies, such as active edible packaging, to maintain the quality and safety of sun-dried snakeskin gourami during storage.

Active edible films and coatings are an emerging food packaging technology that incorporates bioactive compounds into edible biopolymer matrices to enhance multifunctional properties such as antimicrobial, antioxidant, and barrier properties, and extend product shelf life while ensuring food safety [11,12]. Chitosan (CS) and gelatin (GL) biopolymers are widely recognized for their synergistic potential in fabricating edible films [12]. CS is a positively charged, nontoxic, edible, and biocompatible polysaccharide, derived from chitin through deacetylation under alkaline conditions [13]. Its excellent film-forming ability and antimicrobial properties make chitosan one of the most investigated polysaccharides for the fabrication of packaging film [14,15,16]. However, its application as food packaging is often hindered by poor water barrier and mechanical properties [17]. GL, a typical by-product from fish and animal processing, has high proline, glycine, and hydroxyproline contents, resulting in an excellent flexible film-forming ability. GL is a suitable biopolymer for packaging films due to its edibility, biodegradability, abundance, good barrier capacity, and transparency [12,18]. The incorporation of GL reinforces the mechanical robustness and oxygen barrier capacity of CS-based films, offering a promising biocomposite suitable for preventing the lipid oxidation of food materials [19,20,21,22].

Previous studies have examined CS/GL composites, but the utilization of protein hydrolysates derived from fish processing waste as an active component in edible packaging for sun-dried fish products remains largely unexplored. This study bridges this gap by developing an active edible film from a CS/GL composite incorporated with snakeskin gourami waste-derived protein hydrolysate (FPH). The physical and functional properties of the fabricated films were characterized under varying FPH concentrations. Then, the optimal formulation was applied as an edible coating to sun-dried snakeskin gourami to evaluate its efficacy in inhibiting lipid oxidation and maintaining quality parameters under cold storage. This study supports the circular economy and sustainability by transforming industrial waste into a functional packaging solution and also by improving the quality and shelf life of food products, thereby contributing to waste reduction and environmental conservation.

## 2. Materials and Methods

### 2.1. Materials

Chitosan (CS) powder (degree of deacetylation: 75%, viscosity: 5 mPa·s) was purchased from AP Operation Co., Ltd. (Chonburi, Thailand). Fish gelatin (GL) was purchased from Continental Food Co., Ltd. (Bangkok, Thailand). Acetic acid (CH3COOH), alcalase enzyme, 2,2-diphenyl-1-picrylhydrazyl (DPPH), sodium chloride (NaCl), thiobarbituric acid (TBA), trichloroacetic acid (TCA), and Mueller–Hinton agar were purchased from U&V Holding Co., Ltd. (Nonthaburi, Thailand). Neogen Petrifilms were obtained from DKSH Co., Ltd. (Bangkok, Thailand). Heads of snakeskin gourami (*Trichogaster pectoralis*), as the by-products of sun-dried gourami processing, were obtained from a community-based enterprise in Bang Bo District, Samut Prakan Province, Thailand.

### 2.2. Extraction of Fish Protein Hydrolysate (FPH)

The FPH extraction was conducted following Muzaifa et al. [23]. The snakeskin gourami heads were finely ground and mixed with water to achieve a raw material-to-volume ratio concentration of 1.28% (*w*/*v*). The mixture was then heated in a temperature-controlled water bath at 65 °C and 5.76% (*w*/*v*) of alcalase enzyme was added with continuous stirring at 300 rpm. During this process, the pH of the solution was maintained at 7 by regular monitoring every 15 min. The hydrolysis process was continued for 85 min before the enzyme was inactivated by heating at 90 °C for 2 min. The mixture was then cooled, centrifuged at 9000 rpm at 4 °C for 30 min and filtered. FPH was collected as the supernatant and stored at −18 °C until further applications.

### 2.3. Preparation of CS/GL Composite Films Incorporated with FPH (P-CG)

The composite films were prepared following the method reported by Zhang et al. [24] with slight modifications. A 2% (*w*/*v*) CS solution was prepared by dissolving CS powder with distilled water containing 2% (*v*/*v*) acetic acid and heating at 70 °C for 3 h. Similarly, a 2% (*w*/*v*) GL solution was prepared by dissolving GL with distilled water and heating at 50 °C for 2 h. The CS and GL solutions were then combined at a 1:1 (*v*/*v*) ratio and continuously stirred at 50 °C for 30 min to form a homogeneous blend. FPH was then added at 0, 2, 4, 6, and 8% (*v*/*v*) (based on the total weight of CS and GL) to obtain the composite films. The resulting films were designated as CG for the CS/GL composite film and xP-CG for the CS/GL composite films incorporated with FPH (with x referring to the concentration of FPH).

### 2.4. Characterization of P-CG Films

#### 2.4.1. Fourier Transform Infrared (FTIR) Spectroscopy

The chemical structures of the film were characterized using a Fourier transform infrared (FTIR) spectrometer, with composite films scanned over the wavenumber range 4000–400 cm^−1^.

#### 2.4.2. Field Emission Scanning Electron Microscopy (FE-SEM)

Scanning electron microscopy (SEM) analysis of the film was conducted using a Leica 360-S system (Leica Microsystems Ltd., Cambridge, UK) under high vacuum at an acceleration voltage of 15 kV. A thin layer of gold was sputter-coated onto the fractured surface before analyzing to prevent charging during electron beam exposure.

#### 2.4.3. Optical Properties

Film transparency was determined by measuring the absorbance at 600 nm (A600) and calculated using Equation (1) [25]:

(1) Transparency (Abs/mm)=A600/e

where e is the film thickness (mm).

#### 2.4.4. Mechanical Properties

The thickness of the film was measured using a digital micrometer. Tensile strength and elongation at break were determined with a universal texture meter according to the ASTM D882-00 standard method [26]. Before the test, the samples were cut into rectangular strips (60 mm × 20 mm) and conditioned at 23 ± 2 °C and 50% RH for 48 h. The test was conducted using a 40 mm initial grip separation, 0.06 mm/s traction speed, and a 150 kg load cell, with results expressed as mean values calculated from ten replicates.

#### 2.4.5. UV-Barrier Properties

UV-barrier properties were analyzed using UV–Vis spectroscopy following Zhang et al. [24]. The film samples were cut into rectangular strips (4 cm × 1.5 cm) and fixed in quartz spectrophotometer cells. A UV–Vis spectrophotometer (Agilent CARY 100, Varian Corporation, Palo Alto, CA, USA) was used to measure the absorbance at selected wavelengths ranging from 200 to 800 nm.

#### 2.4.6. Antioxidant Properties

The antioxidant activity was determined using the 2,2-diphenyl-1-picrylhydrazyl (DPPH) assay following the method described by Lee et al. [27], with a slight modification. A film sample (20 mg) was added to 40 mL of DPPH solution (0.25 μM in methanol) in a test tube. The mixture was vortexed thoroughly and incubated in the dark at room temperature for 1 h. The absorbance of the supernatant was then measured at 517 nm using a spectrophotometer. The DPPH scavenging activity was calculated using Equation (2):

(2) $$DPPH\;scavenging\;activity\;\left(\%\right)={(A}_{x}-A_{y})/A_{x}\times 100$$

where $A_{x}$ and $A_{y}$ are the absorbance values of the blank solution and the solution after the reaction, respectively.

#### 2.4.7. Antimicrobial Properties

The antibacterial activities of the CG-P film were evaluated using the well diffusion method on Mueller–Hinton agar (MHA) following the method of Jahangirian et al. [28]. *Staphylococcus aureus* (DMST8840) and *Escherichia coli* (DMST4212) were used as microbial references for the antibacterial assay of the CG-P film, and the sizes of the observed inhibition zones were reported in millimeters (mm). The MHA agar plates were inoculated with the bacterial strain under aseptic conditions, and wells (diameter 6 mm) were filled with 50 μL of the respective film-forming solutions and incubated at 37 °C for 24 h.

### 2.5. Application of CG-P Edible Coating for Shelf Life Extension of Sun-Dried Gourami Fish During Cold Storage

#### 2.5.1. Sample Preparation

Whole sun-dried snakeskin gourami, sourced from a community-based enterprise in Bang Bo District, Samut Prakan Province, Thailand was gutted, decapitated, thoroughly washed, and salted.

The sun-dried snakeskin gourami was coated with the CG and CG-P solutions using a spray-coating technique. For each fish, a total volume of 5 mL of the respective coating solution was uniformly applied from head to tail, consisting of two spray passes per side. Following the coating process, the fish were dried in a tray dryer at 40 °C for 3 h. The coated fish samples and sun-dried fish without coating (as a control) were stored in tightly sealed polypropylene (PP) plastic containers at 4 °C under 80% RH before analyzing the quality parameters on days 0, 2, 4, 6, 8, 10, 12, and 14.

#### 2.5.2. Weight Loss

The weight loss of the samples was calculated using the weight difference before and after the test (Equation (3)).

(3) $$Weight\;loss\;(\%)={(W}_{0}-W_{t})/W_{0}\times 100$$

where $W_{0}$ and $W_{t}$ define the initial weight and the weight of samples at time t of storage.

#### 2.5.3. Color Change

The color change in the samples was analyzed using a colorimeter (ColorFlex^®^, Hunter Lab, Reston, VA, USA) to obtain L*, a*, and b*. The total color difference (∆E) values of the samples were calculated using Equation (4):

(4) $$∆E=\;\sqrt{{\left(L^{*}-L_{0}^{*}\right)}^{2}+{\left(a^{*\;}-a_{0}^{*}\right)}^{2}+{\left(b^{*}-b_{0}^{*}\right)}^{2}}$$

where $L_{0}^{*}$, $a_{0}^{*}$, and $b_{0}^{*}$ represent the initial color parameters of the samples.

#### 2.5.4. Thiobarbituric Acid Reactive Substances (TBARS)

Lipid oxidation of the sample was determined by analyzing the TBARS value according to the distillation method [29]. A finely ground sample (10 g) was homogenized with 50 mL of distilled water and distilled with 2.5 mL of 4 M HCl, antifoam agent, and glass beads. A 13 mL aliquot of the distillate was then reacted with 13 mL of 0.2883% TBA reagent in a boiling water bath for 35 min. After cooling in ice water for 10 min, the absorbance was measured at 538 nm. The TBARS value was expressed as mg of malondialdehyde (MDA) per kg of sample and calculated using Equation (5):

(5) TBARS (mg MDA/kg)= 0.78×D/W

where D represents the absorbance difference between the sample and the control, and W is the sample weight (g).

#### 2.5.5. Microbial Analysis

The microbial analysis was conducted according to the Thai Community Product Standard for dried fish (Standard No. 298/2006) [30] using the Petrifilm method. A 25 g aliquot of the sample was mixed with 225 mL of sterilized saline solution (0.85%) in a stomacher bag and homogenized for 2 min using a stomacher. The obtained suspension was then used to perform serial dilutions, with 1 mL dilutions spread on an aerobic count plate (Petrifilm, 3M, St. Paul, MN, USA). The dilutions were incubated at 37 °C for 24–48 h for the analysis of *S. aureus* and *E. coli*, and at 25–30 °C for 3–5 days for the analysis of yeasts and molds. Microbial growth was determined by counting the colony forming units, with results expressed as CFU/g.

#### 2.5.6. Total Volatile Base Nitrogen (TVB-N)

The TVB-N content was measured following the method of Malle and Poumeyrol [31]. The samples (100 g) were homogenized with 200 mL of 7.5% TCA using a high-speed homogenizer for 1 min. The homogenate was then centrifuged at 4000 rpm for 15 min, and 25 mL of the supernatant was transferred into a distillation tube. Then, 6 mL of 10% NaOH was added to the distillation tube, and a beaker containing 10 mL of 4% boric acid with 2–3 drops of an indicator solution (methyl red mixed with bromocresol green) was placed at the outlet of the condenser to capture the distilled volatile bases. The distillation process was continued until the boric acid solution turned green, indicating the presence of alkaline compounds. The collected distillate was then titrated with 0.1 N HCl until the color changed to pale pink. The TVB-N content was calculated using Equation (6):

(6) $$TVB-N=[\left(V-B\right)\times N\times 14]/W\times 100$$

where V is the volume of HCl consumed by the sample (mL), B is the volume of HCl consumed in the blank (mL), N is the concentration of HCl (N), and W is the sample quantity (g). The TVB-N content was reported as mg/100 g of fish meat.

### 2.6. Statistical Analysis

All of the experiments, unless otherwise mentioned, were conducted in triplicate, with data analyzed using SPSS (SPSS 29 for Mac, SPSS Inc., Chicago, IL, USA). Statistical significance between means was calculated using analysis of variance (ANOVA) and Duncan’s new multiple range test at *p* < 0.05.

## 3. Results and Discussion

### 3.1. FTIR Spectroscopy

**Figure 1:**
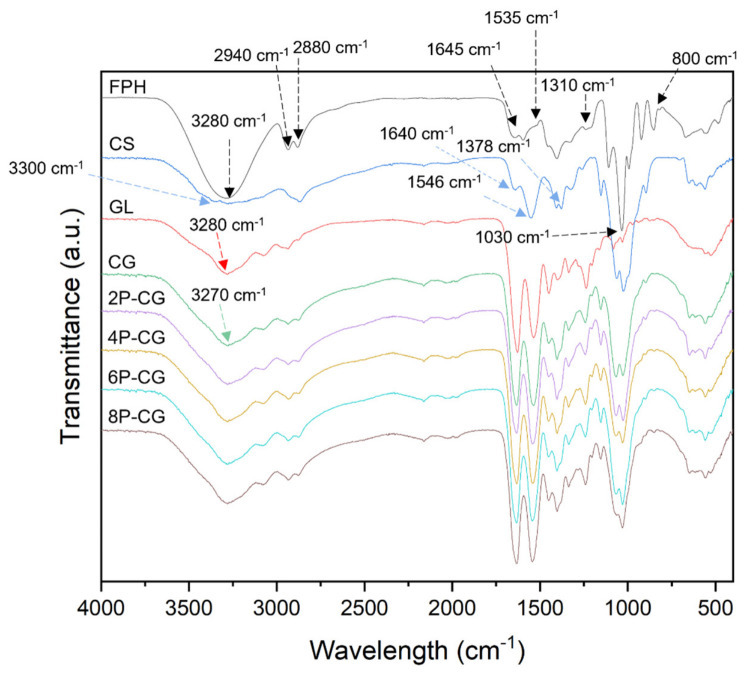
FTIR spectra of CG films incorporated with different concentrations of FPH.

An FTIR analysis was conducted to elucidate the specific chemical structures and intermolecular interactions within the composite films. The FTIR analysis of FPH (Figure 1) exhibited a peak at 3280 cm^−1^, assigned to the amide A band, which is the formation of hydrogen bonding between peptide N–H [32,33,34], with a peak at 2940 cm^−1^ corresponding to the alkyl groups (C–H). Characteristic peaks were observed at 1645 cm^−1^, 1535 cm^−1^, and 1310 cm^−1^, corresponding to the amide I band (C=O stretching vibrations), amide II band (C–N and N–H bending vibrations), and amide III band (C–N stretching vibrations and N–H deformation), respectively, indicating protein secondary structures [35,36,37,38]. The peak at 1030 cm^−1^ represented the C–O bond, and the peak at 800 cm^−1^ indicated C–H bonding in the aromatic rings [39]. The results indicate that FPH was successfully extracted from snakeskin gourami heads.

The FTIR spectra of CS exhibited a broadening peak at 3300 cm^−1^, attributed to O–H and N–H stretching vibrations. Characteristic peaks were also observed at 1640, 1546, and 1378 cm^−1^ contributing to amide I, amide II, and -CH2 bending vibrations, respectively [32,36,40,41]. The GL film spectra showed characteristic peaks at 3280, 1628, 1533, and 1233 cm^−1^, corresponding to amide A, amide I, amide II, and amide III, respectively [32,40,42].

After blending CS and GL, the CG film inherited the characteristic peaks of both polymers. A broadening and shifting of the amide A peak to 3270 cm^−1^ was observed, indicating the formation of intermolecular hydrogen bonding between the carboxyl groups of GL and the amino groups of CS [42,43]. These results confirmed that the composite films were successfully fabricated while still maintaining the functional properties of CS and GL.

All FTIR spectra of CG and P-CGs exhibited similar absorption bands after adding FPH to the CG matrix. The amide A peak (3270 cm^−1^) shifted toward higher wavenumbers as the FPH concentration increased. All films maintained the characteristic peaks of CS, GL, and FPH, despite the addition of protein hydrolysate. The results indicate that FPH was successfully incorporated into the CG matrices without disrupting the existing functional groups.

### 3.2. Morphology

**Figure 2:**
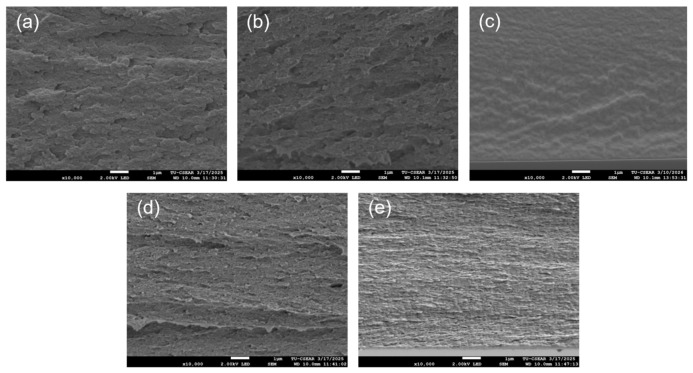
FE-SEM cross-section images of CG films incorporated with different concentrations of FPH. (**a**) CG, (**b**) 2P-CG, (**c**) 4P-CG, (**d**) 6P-CG, and (**e**) 8P-CG.

The morphology of the obtained films was determined by analyzing the cross-section images (Figure 2). The CG films exhibited a uniform and compact morphology (Figure 2a) with minimal visible porosity, suggesting a high degree of compatibility between CS and GL as the matrix and reinforcing phase. The uniformity of the composite was due to the hydrogen bonding that acted as a crosslink between the two polymer chains, forming a polyanion–cation complex [42,44,45]. Adding FPH to the CS matrix significantly increased the uniformity, giving the most compact and smooth structure at 4% FPH (Figure 2b–c). However, the addition of FPH over 4% showed an increase in roughness and phase separation (Figure 2d–e). The deterioration of interfacial adhesion suggests that FPH acted as a structural filler at the optimal concentration, but excessive FPH disrupted the continuity of the polymer matrix [46], causing a negative impact on film integrity.

### 3.3. Optical and UV-Barrier Properties

The transparency values of CG films incorporated with different concentrations of FPH are shown in Table 1. A high transparency value refers to a less transparent film [47]. The CG films exhibited transparency values of 0.90 ± 0.15 Abs/mm. The incorporation of FPH increased the transparency values of the P-CG films, with the highest at 1.23 ± 0.18 Abs/mm in the 8P-CG film. The results indicate that the incorporation of FPH into the CG film matrix decreased the transparency of the composite film, correlating with the morphological images, showing that increased FPH concentration led to surface roughness and phase separation. This could lead to the light scattering effect that makes the film less transparent.

**Figure 3:**
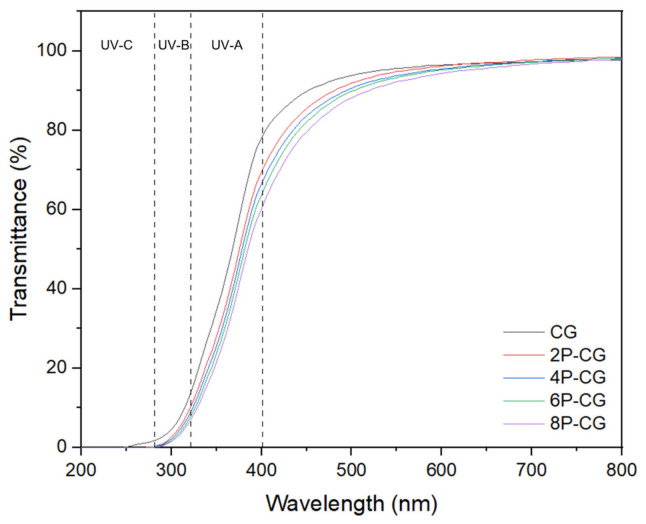
UV–Vis transmittance curves of CG films incorporated with different concentrations of FPH.

The UV-barrier capacity of the films was determined by analyzing light transmittance (%T) at wavelengths of 200 to 800 nm, with lower transmittance reflecting a higher light barrier property. The neat CG film demonstrated effective barrier properties within the UV-B (280–320 nm) and UV-C (100–280 nm) regions (Figure 3), with transmittance values less than 20%. Conversely, at a wavelength of 400 nm, the transmittance of the CG film was 78.1%, indicating high transparency within the UV-A region (320–340 nm) [48]. This optical behavior was attributed to the presence of chromophore molecules in CS and GL that absorbed the UV radiation. In CS, these chromophores included hydroxyl and amino groups, while in GL, they were carboxyl groups from aspartic (Asp) and glutamic (Glu) acid residues, lone electron pairs on nitrogen conjugated with double bonds in histidine (His) and arginine (Arg) residues, hydroxyl groups, and conjugated double bonds within the benzene rings of aromatic amino acids, particularly tyrosine (Tyr) [36,49]. Incorporation of FPH into the CG matrices significantly reduced the transmittance of the P-CG films in the range of 200–400 nm in a concentration-dependent manner, with the lowest transmittance at 60.4% in 8P-CG. The reduction in UV transmittance was due to the presence of aromatic amino acids in the FPH, such as Tyr, tryptophan (Trp), and phenylalanine (Phe), which improved the UV-barrier properties [50,51]. These results suggest that the incorporation of FPH enhanced the UV protection of P-CG films, which may effectively delay lipid oxidation in food products during storage.

### 3.4. Mechanical Properties

The thicknesses of the composite films are presented in Table 1. The thickness of the CG film was 18.00 ± 3.00 μm. Incorporating FPH into the CG matrix increased the film thickness range from 18.00 to 21.00 μm. These results concur with a previous study that reported an increase in the thickness of a CS/GL/whey protein edible film at higher protein concentrations [52].

The tensile strength of the films was also determined (Table 1). The CG film exhibited a tensile strength of 66.45 ± 8.67 MPa. The incorporation of FPH at 2% and 4% increased the tensile strength to 103.30 ± 12.50 MPa and 98.128 ± 12.399 MPa, respectively, with no statistical difference. However, further incorporation of FPH above 4% significantly decreased the tensile strength of the films, with the lowest value for 8P-CG of 52.46 ± 4.79 MPa. In contrast, the CG film exhibited the highest elongation at break at 82.00 ± 0.66%, but this significantly decreased with an increasing concentration of incorporated FPH, reaching the lowest value of 68.42 ± 3.68% in the 8P-CG film.

The results indicate that the incorporation of FPH in the CG matrix affected the mechanical strength of the films. The increase in tensile strength at lower concentrations of FPH was attributed to the formation of hydrogen bonding between the FPH peptides, the amino group of CS, and the carboxyl group of GL, thereby compacting the matrix structure. However, at higher concentrations, over-bonding occurred, resulting in restricting the polymer chain mobility, leading to the loss of flexibility and fragility [53,54,55]. Consistent with the morphology results, the presence of voids and phase separations that occurred after incorporating FPH into the CG matrix may act as stress concentration sites, compromising the mechanical integrity of the films. These results aligned with Ji et al. [56], who reported a decrease in mechanical properties after incorporating protein hydrolysate into the polymer matrix.

### 3.5. Antioxidant Properties

The antioxidant capacity of the films was determined by analyzing their DPPH radical scavenging activity, as presented in Table 2. The CG film exhibited 33.67% radical scavenging activity attributed to inherent CS antioxidant activity. A previous study reported that CS contains tocopherol, which is capable of donating electrons to neutralize free radicals [57]. Incorporating FPH into the CS films significantly enhanced their radical scavenging activity, with the 4P-CG film exhibiting the highest value of 55.57 ± 2.35%. This pronounced increase in antioxidant activity is heavily dictated by the biochemical composition of the protein hydrolysate generated via enzymatic cleavage. During alcalase hydrolysis, large protein structures are broken down into low-molecular-weight peptides. These smaller peptide fractions possess a high concentration of solvent-accessible amino acid residues that act as potent hydrogen or electron donors to stabilize reactive free radicals. Specifically, the presence of hydrophobic amino acids—such as alanine (Ala), valine (Val), leucine (Leu), and isoleucine (Ile)—facilitates greater lipid solubility and interaction with radical species, thereby inhibiting the lipid peroxidation pathways. Concurrently, aromatic amino acids, including tyrosine (Tyr), phenylalanine (Phe), and tryptophan (Trp), contribute to critical proton-donation capabilities. The resonance-stabilized benzene rings of these aromatic residues can effectively donate electrons to electron-deficient radicals without losing their own structural stability. Furthermore, specialized functional groups, such as the imidazole ring in histidine (His) and the nucleophilic thiol group in cysteine (Cys), provide vital protons that neutralize reactive oxygen species (ROS) [4,5]. However, at FPH concentrations above 6%, the radical scavenging activity was significantly reduced. This downward trend is highly correlated with peptide–peptide interactions at elevated concentrations. Excessive peptide loading facilitates molecular crowding and spatial aggregation, which sterically buries the active hydrophobic and aromatic functional groups within the dense core of the clusters. This aggregation restricts the dispersion and availability of these antioxidant groups to react with the DPPH radicals, matching the physical phase separations and increased surface roughness observed in the FE-SEM cross-sectional analysis at higher concentrations (Figure 2) [58]. These findings concur with previous reports indicating that freshwater FPH demonstrates robust, concentration-dependent DPPH radical scavenging profiles up to an optimal threshold [59].

### 3.6. Antimicrobial Properties

The antimicrobial activity was evaluated by measuring the inhibition zone. The CG film exhibited antimicrobial activity against *S. aureus* (Gram-positive) and *E. coli* (Gram-negative). The antibacterial activity of the neat CG film was attributed to the CS amino groups, which interacted with the electronegative charges of the bacterial cell surfaces, leading to leakage of the intracellular components [60]. FPH incorporation increased the inhibition zone of *S. aureus*, but with no observed statistical difference, indicating that the anti-Gram-positive activity is driven predominantly by the base chitosan matrix. In contrast, the inhibition zones against *E. coli* exhibited a significant increase with the concentration of FPH incorporated in the CG films, with the highest inhibition zone observed at 9.03 ± 0.06 in 8P-CG. The composite films demonstrated a mild-to-moderate antibacterial activity, which was enhanced against Gram-negative bacteria due to the biochemical properties of the hydrolyzed peptides. The enzymatic breakdown of snakeskin gourami processing waste releases low-molecular-weight, amphipathic peptides. These specific peptide fractions are rich in positively charged amino acid residues alongside hydrophobic domains. The antimicrobial mechanism operates through a two-step electrostatic and hydrophobic disruption sequence. First, the positively charged residues associate with the electronegative lipopolysaccharide outer membrane of Gram-negative bacteria like *E. coli.* Following this initial binding, the exposed hydrophobic and aromatic amino acid chains insert directly into the non-polar core of the bacterial lipid bilayer. This hydrophobic interaction permeabilizes the cell wall, generating physical pores that induce cytoplasmic leakage, compromise metabolic equilibrium, and inhibit cell proliferation. The relatively thicker peptidoglycan cell wall of *S. aureus* likely acted as a mechanical barrier to these hydrophobic peptide chains, explaining the lack of a statistically significant increase against the Gram-positive strain [4,5,61].

Overall, the incorporation of 4% FPH into the CS/GL matrix was identified as the optimal formulation, providing the best balance of structural integrity and active functional properties required for food preservation. This formulation enhanced the UV-barrier, mechanical, and antioxidant properties of the films while maintaining mild-to-moderate antimicrobial efficacy. These attributes are crucial parameters for delaying food deterioration and enhancing the overall application of active food packaging materials.

### 3.7. Storage Application

**Figure 4:**
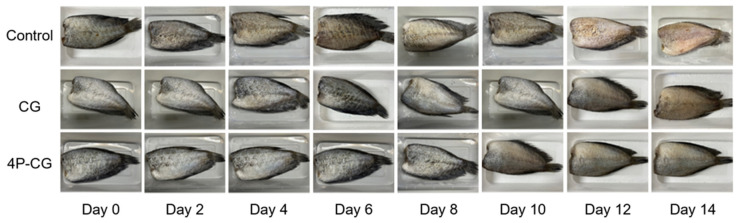
Effect of different coating materials on the visual appearance of sun-dried snakeskin gourami fish for 14 days under cold storage.

**Figure 5:**
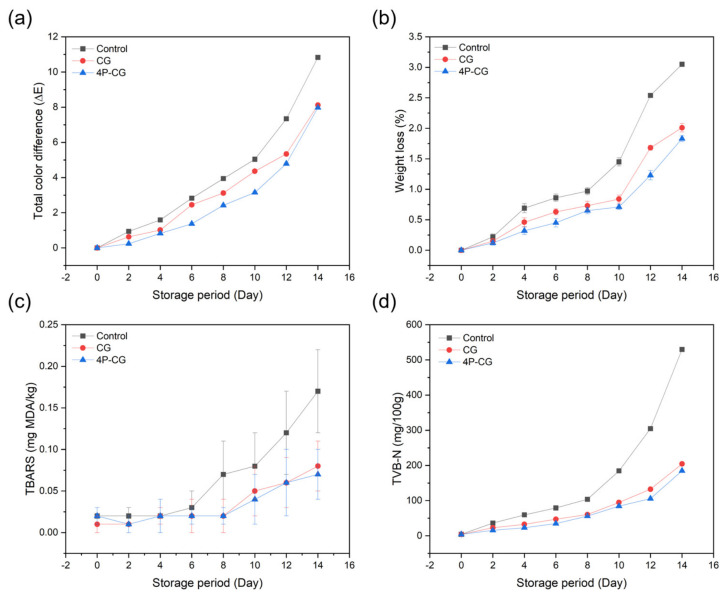
Quality parameters of sun-dried snakeskin gourami coated with the control (without coating), CG, and 4P-CG for 14 days under cold storage. (**a**) ΔE, (**b**) weight loss, (**c**) TBARS, and (**d**) TVB-N.

**Figure 6:**
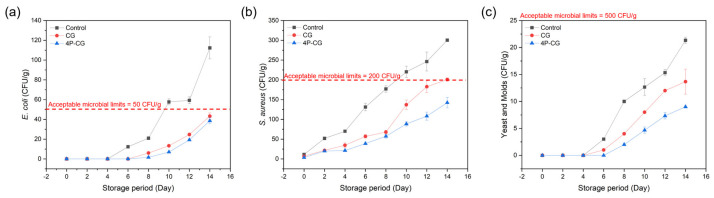
Microbial growth of sun-dried snakeskin gourami coated with the control (without coating), CG, and 4P-CG for 14 days under cold storage. (**a**) *E. coli*, (**b**) *S. aureus*, and (**c**) yeast and molds.

4P-CG was selected as the edible coating on sun-dried snakeskin gourami fish, and the quality parameters were monitored over 14 days during cold storage. The sun-dried fish were coated with 4P-CG and the results were compared with the CG-coated fish and the control (without coating). The visual appearance, change in quality parameters, and the microbial growth of sun-dried snakeskin gourami during storage are shown in Figure 4, Figure 5 and Figure 6, respectively.

As observed, the color of all the sun-dried fish samples changed over storage time (Figure 4), with fish without the coating (control) showing the most significant change, followed by CG and 4P-CG. The total color difference (∆E) was analyzed to confirm the change in color parameters. The ∆E of all samples increased gradually throughout the storage period (Figure 5a). The control exhibited the highest ∆E for all storage periods, reaching the highest value of 10.83 on day 14, while the CG and 4P-CG exhibited a slower increase in E values, reaching 8.12 and 7.98, respectively. This could be attributed to the oxygen-scavenging properties of CS and FPH, which reduce oxidation and inhibit the Maillard reaction, a primary cause of color changes in dried food products [62,63].

The weight loss of the samples is presented in Figure 5b. All samples showed a progressive increase in weight loss over time, with the highest observed in the control sample, reaching 3.05% on day 14. In contrast, the CG- and 4P-CG-coated samples exhibited significantly lower weight loss throughout the storage period of 2.01% and 1.83% on day 14, respectively. The results indicate that the coatings minimized product weight loss. This reduction is attributed to the dense structural network of the CS/GL matrix, which acted as a water barrier, preventing the leakage of water vapor [64]. The incorporation of FPH into the composite film further improved its moisture barrier capacity, thereby efficiently reducing dehydration in the coated sun-dried fish [65].

TBARS analysis is a widely used method for determining lipid oxidation by measuring MDA, a secondary oxidation product that serves as a primary indicator of rancidity in seafood products [29,66,67]. The TBARS values of all samples gradually increased over the storage period (Figure 5c), indicating the progression of lipid oxidation over time. The control sample exhibited the highest increase in TBARS value, rising from 0.02 to 0.17 mg MDA/kg by the end of the storage period. However, the coated samples exhibited a significantly slower rate of TBARS increase compared to the control, with initial values of 0.01 and 0.02 mg MDA/kg increasing to 0.08 and 0.07 mg MDA/kg for CG and 4P-CG, respectively. These results demonstrate that application of the edible coating successfully protected the sun-dried fish from lipid peroxidation. The superior protective effect observed in the 4P-CG coated sample is directly driven by the high concentration of low-molecular-weight peptides and active amino acid residues embedded within the FPH, providing UV-shielding properties and antioxidant activity [50,59]. These findings concur with the antioxidant activity results discussed previously.

TVB-N is an indicator used to assess the freshness and spoilage of aquatic products (Figure 5d). The results showed that the TVB-N value of the control sample increased continuously throughout the storage period, starting at 4.1 mg/100 g and increasing significantly to 529 mg/100 g by day 14. In contrast, the CG and 4P-CG samples exhibited a slower increase in TVB-N levels compared to the control. The CG sample showed an increase from 4.2 mg/100 g to 204 mg/100 g, while the 4P-CG sample had the lowest TVB-N value, increasing from 4.2 mg/100 g to 184 mg/100 g. Considering the TVB-N values, the 4P-CG-coated sample had the longest shelf life, demonstrating its effectiveness in delaying product deterioration. This may be attributed to the antimicrobial properties of CS as a matrix and protein hydrolysate [60,61], which contributed to preserving product freshness.

The microbial spoilage of the product was further determined by the count of bacteria, *E. coli* and *S. aureus*, and yeast and mold populations over time. The Thai community Product Standard states that the acceptable microbial limits for dried fish must not exceed 50, 200, and 500 CFU/g for *E. coli*, *S. aureus*, and yeasts and molds, respectively. Figure 6a–c shows that all samples exhibited microbial growth over the storage period. The highest was observed in the control, followed by a significant reduction in CG and 4P-CG. For the growth of *E. coli* (Figure 6a), the control sample exceeded the acceptable microbial limit on day 10, reaching 57 CFU/g, and continued to increase to 104 CFU/g by day 14. In contrast, the CG and 4P-CG samples exhibited *E. coli* growth, but remained within the acceptable limits until day 14, with counts of 47 CFU/g and 38 CFU/g, respectively. Similarly, for *S. aureus* (Figure 6b), the growth in the control sample exceeded the standard limit on day 10 with a count of 214 CFU/g. The CG sample also surpassed the growth limit on day 14 with a count of 204 CFU/g. However, the growth of *S. aureus* in the 4P-CG sample remained within the acceptable range until day 14, with a count of 148 CFU/g. On the other hand, despite all samples exhibiting an increasing trend in yeast and mold counts (Figure 6c), the value remained within the acceptable range throughout the study. These results indicate that the application of composite film coatings delayed microbial proliferation compared to the control and extended the shelf life of the samples.

Overall, the results demonstrate that the application of 4P-CG as an edible coating extended the shelf life of sun-dried snakeskin gourami fish by prolonging its critical quality attributes for 14 days under cold storage. Its efficacy in maintaining the storage qualities of the sun-dried fish suggests high potential for the preservation of a broad range of fatty aquatic products susceptible to oxidative and microbial deterioration.

## 4. Conclusions

In this study, an active edible film based on CS and GL incorporating FPH extracted from fish processing waste was successfully developed. The incorporation of FPH into CG films significantly enhanced the mechanical strength, antioxidant capacity, and antimicrobial efficacy of the films. These property improvements were concentration-dependent, but adverse effects on mechanical and antioxidant properties were observed in FPH concentrations exceeding 4%. Therefore, the 4P-CG film was identified as the optimal formulation and used to study the storage application of sun-dried snakeskin gourami fish under cold storage. Application of the 4P-CG coating significantly improved the storage stability by reducing weight loss, delaying lipid oxidation, and inhibiting microbial proliferation, effectively extending the product’s shelf life by four days compared to the control and neat CG-coated fish. This study provides a sustainable, circular-economy-driven solution by transforming industrial by-products into a high-value active packaging system. Our findings offer a promising strategy to reduce the environmental impact and minimize food waste, while ensuring the safety and quality of fatty aquatic products that are susceptible to oxidative and microbial deterioration.

## Figures and Tables

**Table 1:** Optical and mechanical properties of CG films incorporated with different concentrations of FPH *.

Sample	Transparency (Abs/mm)	Thickness (μm)	Tensile Strength (MPa)	Elongation at Break (%)
CG	0.90 ± 0.15 ^b^	18.00 ± 3.00 ^b^	66.45 ± 8.67 ^c^	82.00 ± 0.66 ^a^
2P-CG	0.85 ± 0.09 ^b^	20.00 ± 2.00 ^ab^	103.30 ± 12.50 ^a^	76.88 ± 2.69 ^b^
4P-CG	1.12 ± 0.13 ^ab^	18.00 ± 2.00 ^b^	98.13 ± 12.40 ^a^	70.92 ± 4.08 ^c^
6P-CG	1.03 ± 0.15 ^ab^	21.00 ± 3.00 ^a^	82.87 ± 4.50 ^b^	69.46 ± 2.50 ^c^
8P-CG	1.23 ± 0.18 ^a^	21.00 ± 3.00 ^a^	52.46 ± 4.79 ^d^	68.42 ± 3.68 ^c^

* Data represent mean values ± SD. Superscripts a–d in columns do not vary significantly (*p* < 0.05) with respect to each other.

**Table 2:** Antioxidant and antimicrobial properties of CG films incorporated with different concentrations of FPH *.

Sample	DPPH Scavenging Activity (%)	Inhibition Zone (mm)	
		*S. aureus*	*E. coli*
CG	33.67 ± 2.46 ^c^	9.03 ± 0.05 ^a^	8.60 ± 0.17 ^b^
2P-CG	44.36 ± 1.04 ^b^	9.03 ± 0.15 ^a^	8.67 ± 0.29 ^ab^
4P-CG	55.57 ± 2.35 ^a^	9.17 ± 0.15 ^a^	8.80 ± 0.26 ^ab^
6P-CG	44.86 ± 1.65 ^b^	9.13 ± 0.12 ^a^	8.93 ± 0.06 ^ab^
8P-CG	41.96 ± 2.24 ^b^	9.20 ± 0.10 ^a^	9.03 ± 0.06 ^a^

* Data represent mean values ± SD. Superscripts a–c in columns do not vary significantly (*p* < 0.05) with respect to each other.

## Data Availability

The original contributions presented in this study are included in the article. Further inquiries can be directed to the corresponding author.
